# THINK surgical TSolution-One^®^ (Robodoc) total knee arthroplasty

**DOI:** 10.1051/sicotj/2017052

**Published:** 2017-10-30

**Authors:** Ming Han Lincoln Liow, Pak Lin Chin, Hee Nee Pang, Darren Keng-Jin Tay, Seng-Jin Yeo

**Affiliations:** 1 Department of Orthopaedic Surgery, Singapore General Hospital 20 College Road, Academia, Level 4 Singapore 169865; 2 The Orthopaedic Centre, Mount Elizabeth Medical Centre #08-02 3 Mount Elizabeth Singapore 228510

**Keywords:** Robotic-assisted, Robot-assisted, Total knee arthroplasty, TKA, THINK surgical, TSolution-One, Robodoc

## Abstract

THINK Surgical TSolution-One^®^ is an active-autonomous, image-based, robotic milling system which enables the surgeon to attain a consistently accurate implant component positioning. The TSolution-One^®^ system is capable of achieving this through an image-based preoperative planning system which allows the surgeon to create, view and analyse the surgical outcome in 3D. The accuracy and precision of component positioning have been attributed to the following factors: customized distal femoral resection, accurate determination of the femoral rotational alignment, minimization of errors and maintenance of bone temperature with robotic milling. Despite all these advantages, there is still a paucity of long-term, high-quality data that demonstrates the efficacy of robotic-assisted total knee arthroplasty (TKA). Questions regarding radiation risks, prolonged surgical duration and cost-effectiveness remain unanswered. This paper aims to describe: (1) TSolution-One^®^ surgical technique; (2) limitations and complications; (3) clinical and radiological outcomes.

## Introduction

The Robodoc system (Curexo Technology, Fremont, CA) was the first robotic system to be used in Orthopaedic surgery in 1992 [1]. Subsequently, Curexo Technology Corporation changed its name to THINK Surgical Inc. (Fremont, CA) in September 2014, renaming Robodoc as TSolution-One^®^. TSolution-One^®^ is an active-autonomous, image-based, robotic milling system which is capable of reproducing accurate component placement and an ideal hip-knee-ankle (HKA) mechanical axis (MA), through an image-based preoperative planning system which allows the surgeon to create, view and analyse the surgical outcome in 3D [2]. This ability to have a surgical endpoint prior to the surgery is a unique capability of orthopaedic robotic-assisted surgery [3].

Restoration of the MA within ± 3° has been reported to be associated with better clinical outcomes and implant survivorship [4–6]. TSolution-One^®^ has been shown to achieve reduction of MA outliers through precise and accurate component implantation [7] which have been attributed to customized distal femoral resection [8], accurate determination of the femoral rotational alignment [9], minimization of human errors associated with oscillating saw [7, 10] and maintenance of bone temperature with robotic milling [11, 12].

Despite these advantages, there is still a paucity of long-term, high-quality data that demonstrates the efficacy of TSolution-One^®^ total knee arthroplasty (TKA). Majority of existing literature has demonstrated improvements in radiological outcomes with no significant differences in functional scores [2, 13, 14], with only one study reporting subtle improvements in health-related quality-of-life (HRQoL) measures in TSolution-One^®^ patients at short-term follow-up [15]. This paper aims to describe: (1) TSolution-One^®^ surgical technique; (2) limitations and complications; (3) clinical and radiological outcomes.

## TSolution-One^®^ surgical technique

Indications for robotic-assisted TKA using the TSolution-One^®^ system are similar to conventional TKA. Ideal patients should be > 60 years, have body mass index (BMI) < 25 kg/m^2^, end-stage osteoarthritis, mild to moderate coronal deformity, a fixed flexion deformity < 15° and intact neurovascular status of the affected limb. Relative contraindications include obese patients with severe coronal deformity > 15°, fixed flexion deformity > 15°, inflammatory arthropathy and ligamentous laxity.

**Figure 1.**
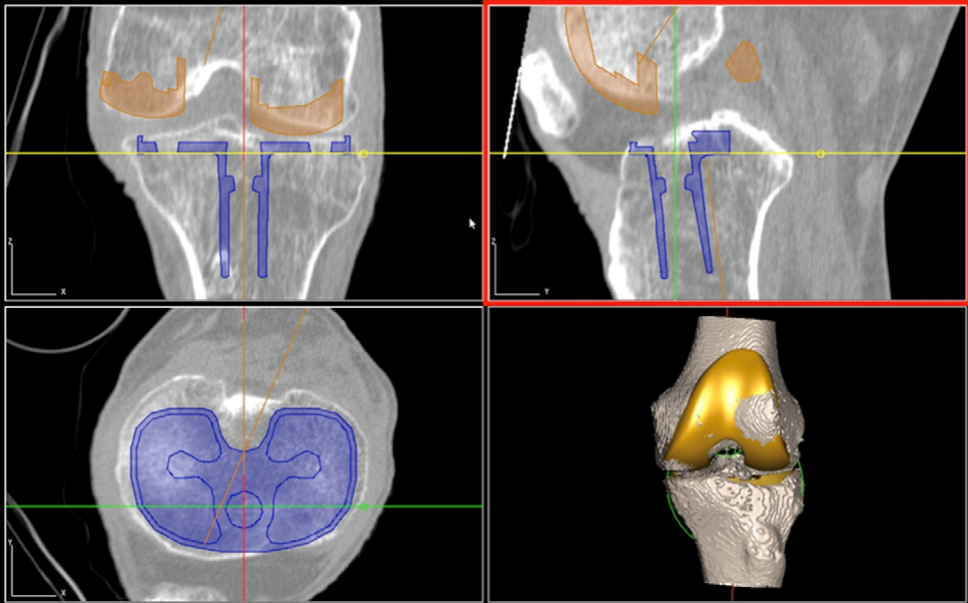
Virtual surgery conducted using TPLAN^®^ 3D workstation.

Preoperative radiography (anteroposterior, lateral, skyline, long-leg films) and computed tomography (CT) of the affected lower limb are performed. A fine-cut (< 3 mm) CT scan is essential for the preoperative “virtual surgery”. The CT images are imported into the TPLAN^®^ 3D planning workstation (THINK Surgical, Inc., Fremont, CA) for image-based preoperative planning (Figure 1). TSolution-One^®^ is an “open” platform which allows the surgeon to select virtual femoral and tibial implants based on the type/size of implant required (posterior-stabilized/cruciate-retaining). The virtual implants are matched onto the surface models to attain a virtual HKA axis of 180° with a sagittal, posterior tibial slope in accordance with prosthesis manufacturer’s instrumentation guide. Femoral component rotation is parallel to the transepicondylar axis. Tibial component rotation in the axial plane is based off the posterior cruciate insertion point and a point marking the medial 1/3 width of the tibial tuberosity. Time taken for “virtual surgery” is approximately 15–20 minutes.

**Figure 2.**
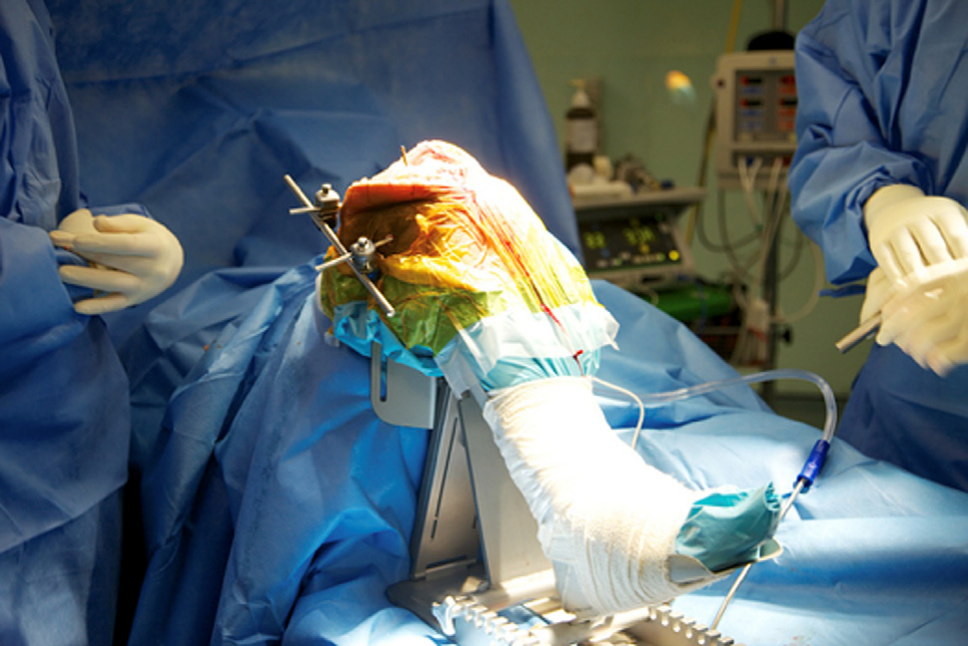
Customized foot and thigh holder.

**Figure 3.**
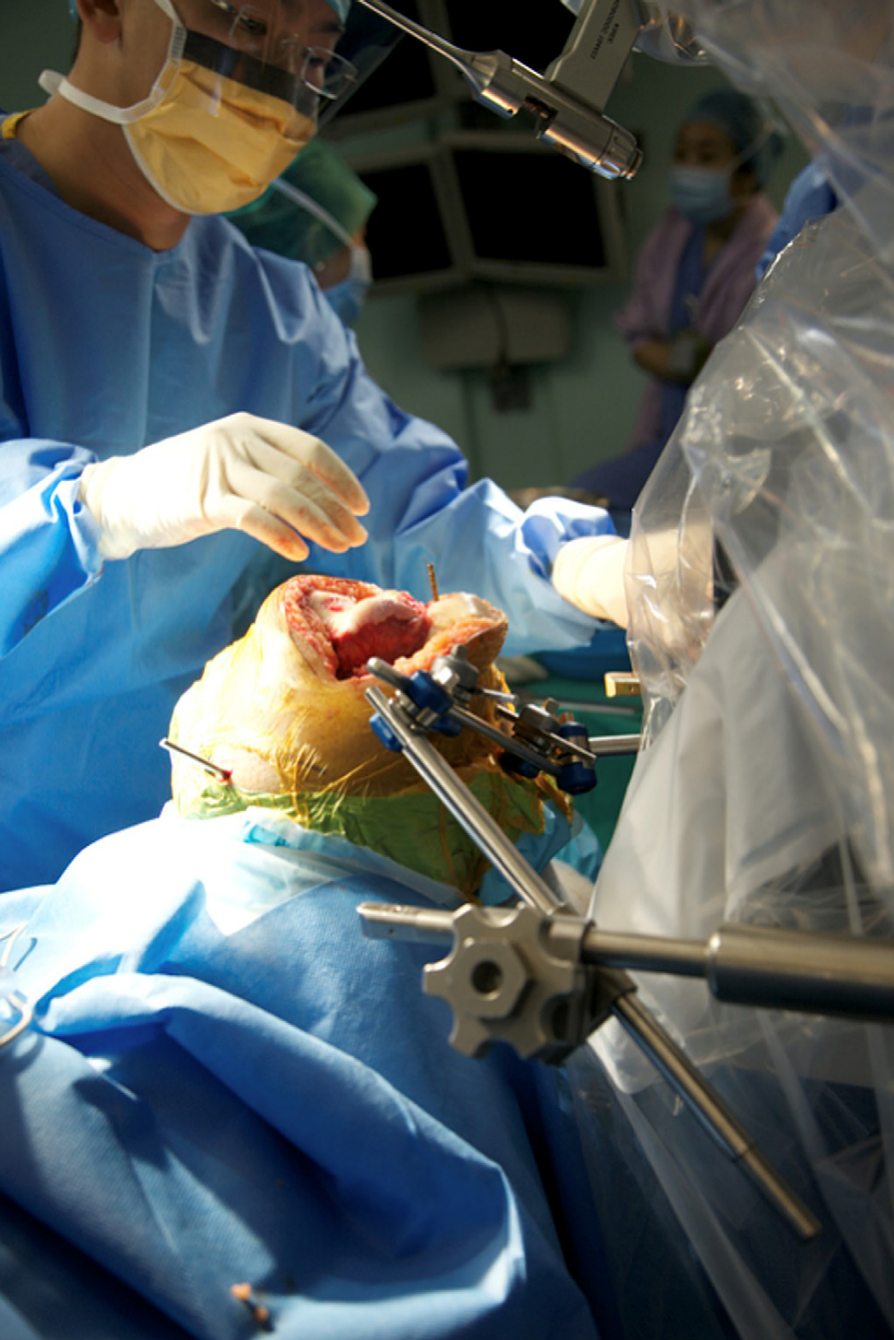
Rigid mating of the patient to TCAT^®^.

**Figure 4.**
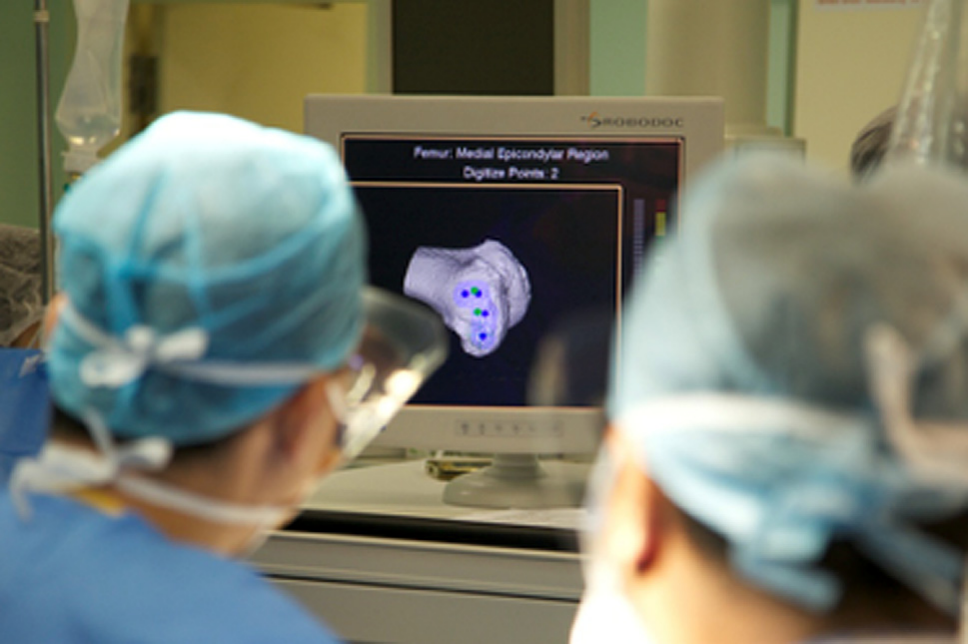
Digitization of femoral landmarks.

**Figure 5.**
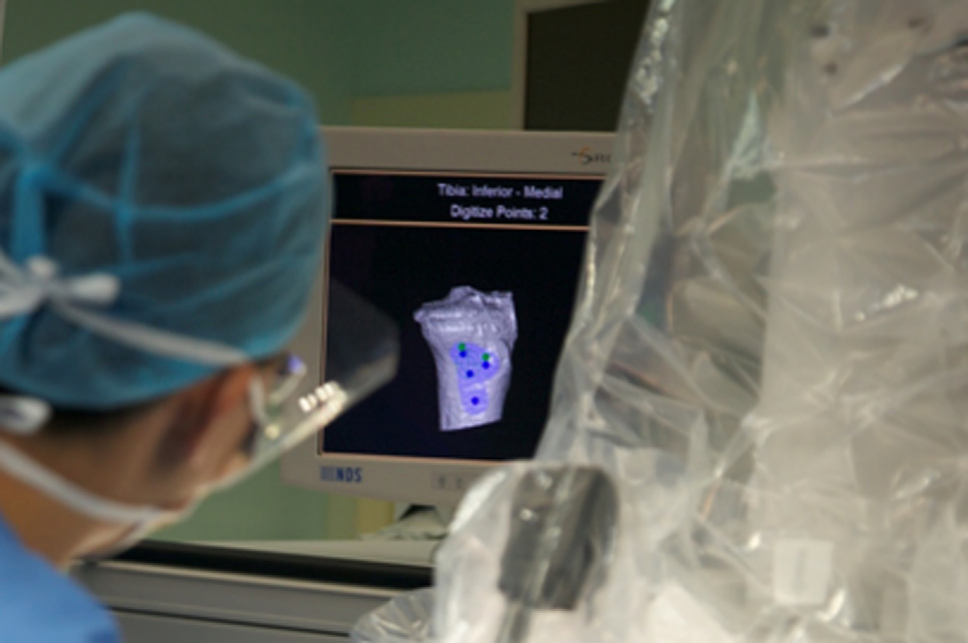
Digitization of tibial landmarks.

This preoperative image-based plan is uploaded to TCAT^®^ robotic-assisted tool prior to surgery. TCAT^®^ is draped and prepared in a sterile manner. A thigh tourniquet is applied and the leg is fixed using a custom foot holder and thigh support (Figure 2). A standard medial parapatellar approach with patella eversion is performed. Stabilization pins, navigation markers and bone movement monitors are placed. Workspace checks are conducted prior to rigid mating of the patient to TCAT^®^. Upon completion of workspace checks, the patient is rigidly connected to TCAT^®^ via two transverse stabilization pins in the distal femur and proximal tibia which are subsequently connected to a special fixation frame linked to TCAT^®^ (Figure 3). The surgeon will identify anatomic landmarks on the femur (Figure 4) and the tibia (Figure 5) and digitize these points as part of the registration process. Upon completion, TCAT^®^ will match the preoperative TPLAN^®^ 3D image-based plan with the intraoperative registration, thereby formulating a milling workspace for the femur and tibia in three-dimensional space.

**Figure 6.**
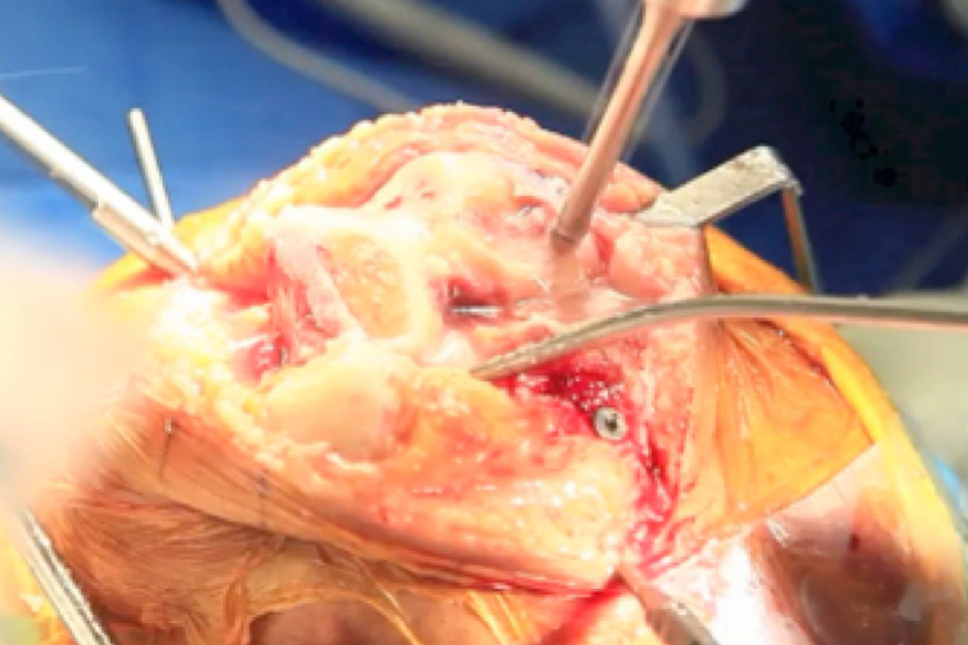
TCAT^®^ miller working on femur.

The surgeon activates TCAT^®^ robotic-assisted tool which proceeds to complete all femoral and tibia bone cuts via the robotic miller (Figure 6). The surgeon maintains control over the milling cutter via a manual override safety button. This process is aided by constant water irrigation for cooling and the removal of milling debris. Soft tissue balancing and a trial of the predetermined femur and tibia components are performed once the milling process is completed. Finalized components are cemented, and stability, patellar tracking and range of motion were assessed. Patella can be selectively resurfaced based on the degree of cartilage wear. An intrasynovial and intramuscular analgesic injection is given if there are no contraindications. Wound closure is performed in a routine fashion via layered closure. Postoperatively, all patients received standard mechanical and pharmacologic thromboprophylaxis. Rehabilitation in accordance with the integrated care pathway was prescribed. Weight-bearing radiographies (anteroposterior, lateral, skyline, long-leg films) are performed at the specialist outpatient clinic at one-month follow-up.

## Limitations and complications of robotic-assisted TKA

The robotic-assisted TKA can be performed by one surgeon, one assistant and one scrub nurse. One additional THINK Surgical staff is required on-site to control the robot, provide technical assistance and rectify intraoperative workspace errors as required. Workspace-related errors are common for TSolution-One^®^ and occur when TCAT^®^ perceives that the knee is outside the miller’s working range. Related to workspace errors, the lack of intraoperative versatility while using TSolution-One^®^ results in abandonment and conversion to a conventional procedure, resulting in time and monetary losses, as well as unnecessary radiation [16, 17]. Such cases have been reported to be as high as 22% and it is essential to understand, anticipate and prevent such occurrences [18]. Attention to proper positioning of the patient and TCAT^®^ prior to rigid coupling and strict compliance with TCAT^®^ workspace checks will reduce such errors. In relation to this, performing a robotic-assisted TKA has a gradual learning curve, as the procedure primarily differs in patient positioning, robot-patient coupling and registration process. The first 15 cases had longer tourniquet times, with a mean of 10 min, attributed to familiarization with the new robot-assisted surgical technique. In addition, the time taken for the robot to mill the femur and tibia is longer than using a conventional saw and this adds additional time to the procedure. Tourniquet time was reduced to a mean of 91 min once surgeons were familiar with the technique.

The current system performs planned cuts within the predetermined 3D workspace and does not possess the ability to differentiate between difference tissue types. This requires the surgeon to move soft tissues away from the path of the miller or stop TCAT^®^ to prevent damage to soft tissues during the procedure. New system updates need to address the possibility of soft tissue detection to prevent iatrogenic injury and the capability to perform soft tissue balancing. In addition, it should allow modification of the preoperative plan to allow for contingencies [19].

**Figure 7.**
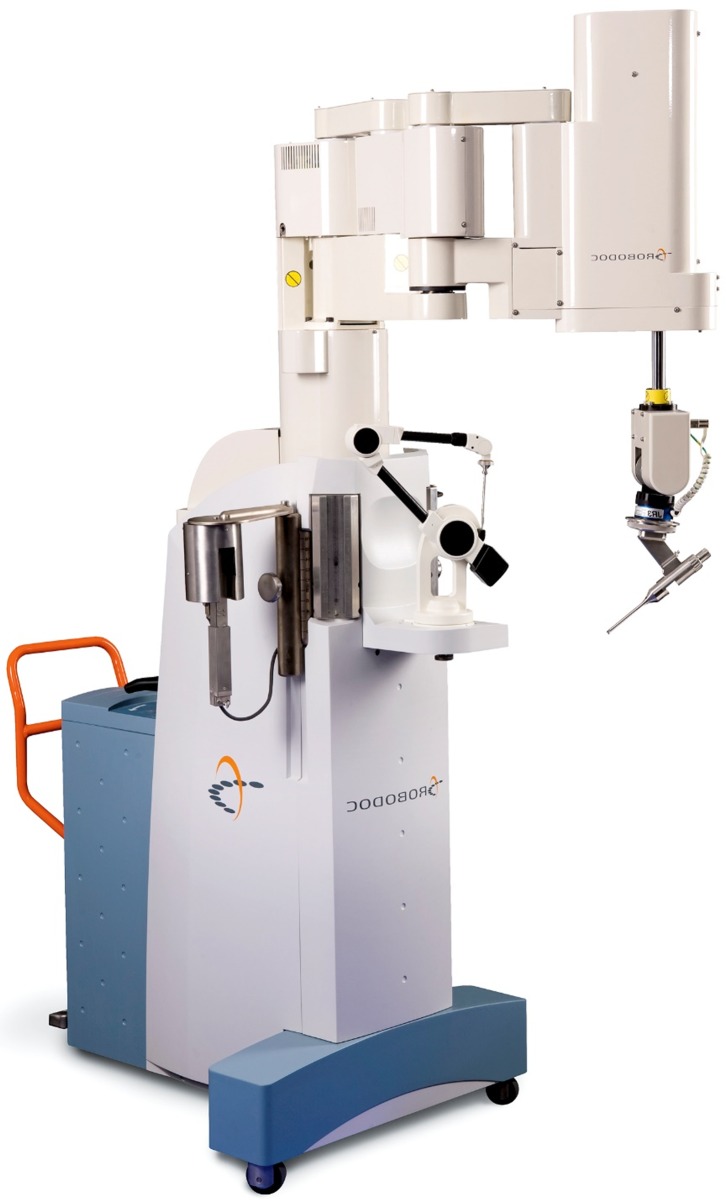
TSolution-One^®^ (Robodoc) system.

TSolution-One^®^ TKA has not been proven to be cost-effective due to the lack of long-term survivorship and outcome data. Cost and regulatory hurdles (government and insurance companies) continue to resist adoption of expensive, new technology which has not demonstrated definite cost-effectiveness [20]. Procuring a TSolution-One^®^ system (Figure 7) does not guarantee better outcomes nor a return of the investment. A high capital (USD 800,000) and recurring cost per patient (USD 1500) are required to operate a robotic surgical system in our institution. Interestingly, it has been reported that arthroplasty centres with robotics may experience greater market growth when compared to centres without robotics [21]. Moschetti et al. used a Markov decision analysis model to demonstrate that robotic-assisted unicompartmental knee arthroplasty is more cost-effective than conventional surgery [22]. The history of robotics has shown that cost-effectiveness of robotic-assisted procedures will continue to improve and enable it to become mainstream.

TSolution-One^®^ is an open platform which allows different manufacturer implants to be used in accordance with surgeon’s preference or patient’s individualized needs. Open platforms such as TSolution-One^®^ provide surgeons with the convenience of inbuilt 3D implant data for multiple designs but may theoretically lack the depth of biomechanical kinematic data present in closed platforms which use proprietary implants.

There were certain complications associated with robotic-assisted TKA at our institution. It was noted that 3.2% of our patients required revision surgery for acute hematogenous periprosthetic joint infection. Robotic-assisted total hip arthroplasty using the previous generation robot resulted in higher infection rates than conventional procedures [23]. However, the risk of infection in robotic-assisted TKA is uncertain. Today, the risk of revision for periprosthetic joint infection (PJI) in modern knee arthroplasty implants is 2.0% based on registry data [24], which is close to the 3.2% at our institution. Increased operating time has been correlated with an increased risk of infection and an operative duration of 127 min for a TKA procedure has been described as a critical operative duration in terms of infection risk [25]. The mean surgical duration for our robotic-assisted TKA patients was 91 min. This was similar to Song et al.’s study (90–100 min) [14]. In addition, when we reviewed the timing of the infections [26], our patients likely suffered from acute hematogenous PJI. It is unlikely that the robotic-assisted TKA PJI was related to prolonged operative time at our institution. As many as 3.2% of patients required early revision surgery due to technical errors secondary to robotic procedure abandonment. For example, one patient had an excessively lateralized tibial component after the robotic procedure was abandoned, which required revision of the tibial component at 20 months. Of note, the robotic-assisted TKA procedure was aborted in 10% of our patients. Separately, 6.5% of patients in the robotic-assisted group developed postoperative soleal vein thrombosis. This may have been related to rigid positioning of the limb during robotic surgery, highlighting the importance of sufficient padding when applying the robotic surgical leg holder. No pin-site related complications or patellar tendon abrasions resulted from the use of robotic-assisted TKA.

## Radiological and clinical outcomes

Robotic-assisted TKA has demonstrated clinical success and excellent radiological outcomes. Multiple studies comparing TSolution-One^®^ TKA and conventional TKA have reported 0% MA outliers in the robotic-assisted group [2, 7, 14]. Kim et al. recently demonstrated good radiological and clinical outcomes of TSolution-One^®^ TKA in end-stage haemophilic arthropathy with severe bony deformity and destruction [27]. In terms of radiologic outcomes, there were no coronal plane mechanical axis outliers (defined as malalignment > 3°) in our robotic-assisted TKA patients and 19.4% (*p* = 0.05) outliers in the conventional control group. In addition, the robotic-assisted patients had significantly less joint line shift outliers (> 5 mm deviation, 3.2% vs. 20.6%) and anterior femoral notching cases (0% vs. 10.3%) when compared to the conventional patients.

However, there is a paucity of long-term clinical outcomes of TSolution-One^®^ TKA, with short and mid-term studies demonstrating no significant difference in functional outcomes when compared to conventional TKA. Park and Lee compared outcomes of robotic-assisted TKA with conventional knees and reported no differences in Knee Society Scores at a mean follow-up of four years [28]. Interestingly, Song et al. reported higher but non-statistically significant Hospital for Special Surgery Knee Score (HSS) and Western Ontario and McMaster Universities Osteoarthritis Index health-related quality-of-life (WOMAC HRQoL) scores in the TSolution-One^®^ cohort in two studies [13, 14]. Similarly, at two years follow-up, we found significant differences in outcome scores for several 36-Item Short Form Survey (SF-36) parameters (general health, vitality and role-emotional) and a trend towards higher functional scores in our robotic-assisted TKA patients when compared to the conventional patients, albeit non-significant [15]. In addition, a significantly larger percentage of robotic-assisted TKA patients attained Minimal Clinically Important Difference (MCID) in SF-36 vitality. This was accompanied by a higher percentage of robotic-assisted patients who achieved MCID in SF-36 QoL scores. This may represent an early indication of improved functional outcomes associated with accurate joint alignment restoration after robotic-assisted TKA.

## Conclusion

In conclusion, robotic TKA technology will continue to grow and revolutionize orthopaedic surgery. Future innovations include improvements of robotic TKA workflow, advanced intraoperative gap-balancing sensors, new biomimetic implant designs which can replicate pre-arthritic knee kinematics and robotically controlled instrumentation with soft tissue balancing. Today, robot-assisted TKA consistently improves overall mechanical alignment and reduces variability with some emerging evidence supporting improvements in clinical outcomes. We are at the “preindustrial” phase of the robotic surgical evolution and we predict that it will gradually become an indispensable adjunct to the orthopaedic surgeon, allowing optimization of patient-specific arthroplasty.

## Conflict of interest

All authors certify that they have no financial conflict of interest (e.g., consultancies, stock ownership, equity interest, patent/licensing arrangements, etc.) in connection with this article.
